# Comparing the Accuracy of Two Generated Large Language Models in Identifying Health-Related Rumors or Misconceptions and the Applicability in Health Science Popularization: Proof-of-Concept Study

**DOI:** 10.2196/63188

**Published:** 2024-12-02

**Authors:** Yuan Luo, Yiqun Miao, Yuhan Zhao, Jiawei Li, Yuling Chen, Yuexue Yue, Ying Wu

**Affiliations:** 1 School of Nursing, Capital Medical University, 10 Xitoutiao, Youanmen Wai, Fengtai District, Beijing, 100069, China, 86 10839117; 2 School of Nursing, Johns Hopkins University, Baltimore, MD, United States

**Keywords:** rumor, misconception, health science popularization, health education, large language model, LLM, applicability, accuracy, effectiveness, health related, education, health science, proof of concept

## Abstract

**Background:**

Health-related rumors and misconceptions are spreading at an alarming rate, fueled by the rapid development of the internet and the exponential growth of social media platforms. This phenomenon has become a pressing global concern, as the dissemination of false information can have severe consequences, including widespread panic, social instability, and even public health crises.

**Objective:**

The aim of the study is to compare the accuracy of rumor identification and the effectiveness of health science popularization between 2 generated large language models in Chinese (GPT-4 by OpenAI and Enhanced Representation through Knowledge Integration Bot [ERNIE Bot] 4.0 by Baidu).

**Methods:**

In total, 20 health rumors and misconceptions, along with 10 health truths, were randomly inputted into GPT-4 and ERNIE Bot 4.0. We prompted them to determine whether the statements were rumors or misconceptions and provide explanations for their judgment. Further, we asked them to generate a health science popularization essay. We evaluated the outcomes in terms of accuracy, effectiveness, readability, and applicability. Accuracy was assessed by the rate of correctly identifying health-related rumors, misconceptions, and truths. Effectiveness was determined by the accuracy of the generated explanation, which was assessed collaboratively by 2 research team members with a PhD in nursing. Readability was calculated by the readability formula of Chinese health education materials. Applicability was evaluated by the Chinese Suitability Assessment of Materials.

**Results:**

GPT-4 and ERNIE Bot 4.0 correctly identified all health rumors and misconceptions (100% accuracy rate). For truths, the accuracy rate was 70% (7/10) and 100% (10/10), respectively. Both mostly provided widely recognized viewpoints without obvious errors. The average readability score for the health essays was 2.92 (SD 0.85) for GPT-4 and 3.02 (SD 0.84) for ERNIE Bot 4.0 (*P*=.65). For applicability, except for the content and cultural appropriateness category, significant differences were observed in the total score and scores in other dimensions between them (*P*<.05).

**Conclusions:**

ERNIE Bot 4.0 demonstrated similar accuracy to GPT-4 in identifying Chinese rumors. Both provided widely accepted views, despite some inaccuracies. These insights enhance understanding and correct misunderstandings. For health essays, educators can learn from readable language styles of GLLMs. Finally, ERNIE Bot 4.0 aligns with Chinese expression habits, making it a good choice for a better Chinese reading experience.

## Introduction

In modern society, rapid information dissemination has given rise to a range of issues, one of which is health-related rumors and misconceptions [1]. When people misunderstand health information, these misconceptions are continually propagated, making it easy for rumors to arise [2,3]. These misconceptions can mislead people to make harmful decisions due to a lack of accurate guidance [2,4,5]. Notably, the general public often lacks scientific knowledge about health information, posing a challenge in distinguishing rumors and misconceptions from truth. Meanwhile, the endogenous health information demand of the public stimulates its dissemination by mass media while providing opportunities for rumor mongers to spread rumors [6]. Up to now, official media or institutions have been the main force in debunking rumors and misconceptions. However, relying on official institutions for the refutation has limitations. The lack of medical and health expertise in official media or institutions leads to a slow official response to unexpected public health issues [7]. Over time, the delayed response can undermine public trust and confidence [8].

Health workers have the responsibility to undertake health science popularization work, so as to change the public’s misconceptions. However, in practical work, the effect of traditional health education patterns is limited. The emergence of generated large language models (GLLMs) has opened up new possibilities for the identification of health-related rumors. In previous studies, GLLMs can be used to create debunking messages against health-related rumors [9,10]. In China, Baidu developed the Enhanced Representation through Knowledge Integration Bot (ERNIE Bot) in 2019. Now, GPT-4 (OpenAI) and ERNIE Bot 4.0 represent the most recent advancements, having undergone significant enhancements in their capabilities [11,12]. Previous studies have found that ChatGPT could provide relatively precise health information in the English context [13,14]. However, health-related rumors and misconceptions may exhibit different characteristics across different languages and cultural backgrounds, posing higher demands on the generalization capabilities of the models. Mandarin is one of the most widely spoken languages in the world, making it crucial to examine the effectiveness of GLLMs within its linguistic context. Such exploration could potentially lead to improvements in the cultural sensitivity of models, thereby enhancing their utility for a wide range of applications in the Chinese-speaking community. Therefore, we conducted a proof-of-concept study to compare GPT-4 and ERNIE Bot 4.0 within the Chinese context. The aims were to explore the accuracy of identifying health-related rumors or misconceptions by GPT-4 and ERNIE Bot 4.0 and to further evaluate the applicability of health science popularization essays generated by them.

## Methods

### Ethical Considerations

This study aimed to compare the accuracy of rumor identification and the effectiveness of health science popularization between GPT-4 and ERNIE Bot 4.0, which did not involve the recruitment of human participants. We used health-related rumors, misconceptions, and truths sourced from publicly accessible web-based platforms to test GPT-4 and ERNIE Bot 4.0. The requirement for a formal ethical review was waived. In this study, all researchers, data collectors, and evaluators were aware of the research objectives and conducted the research in accordance with the study design. This study posed no harm to them.

### Materials and Designs

**Figure 1.**
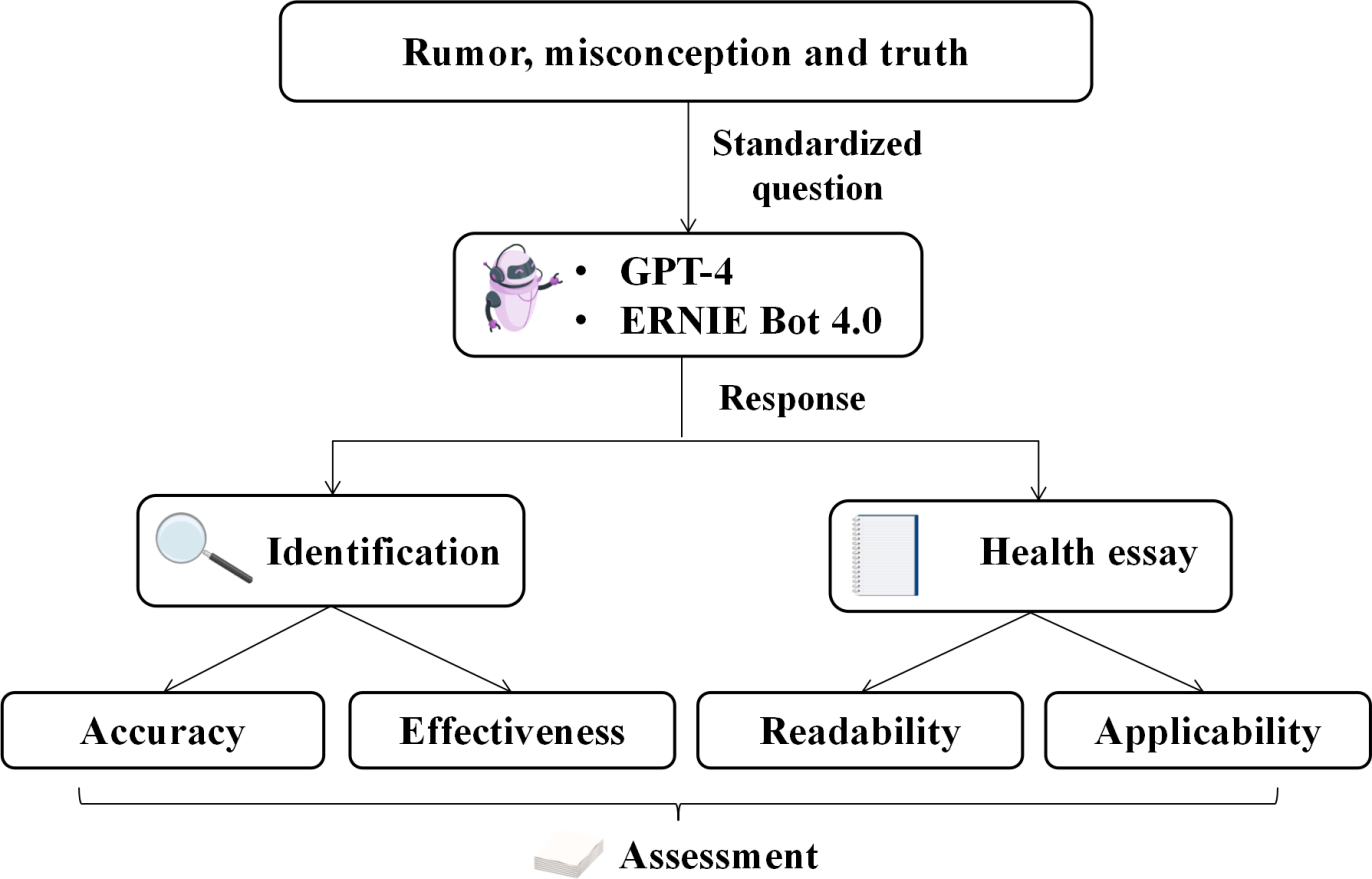
The study process. ERNIE Bot: Enhanced Representation through Knowledge Integration Bot.

The health-related rumors, misconceptions, and truths were selected from the China Internet Disinformation Platform (CIDP). The inclusive materials were issued from January 1 to December 10, 2023. Based on previous proof-of-concept studies, a sample size of 30 is required for feasibility testing [15,16]. Considering the primary objective was to investigate the identification capabilities, the ratio of rumors and misconceptions to truths was 2:1. By using a 2:1 ratio, we aimed to ensure that rumors, which are often more prevalent and varied in web-based environments, were adequately represented in our sample. Considering practical constraints such as time and resource limitations, the 2:1 ratio allowed us to manage these constraints while still achieving a representative and meaningful dataset for our research objectives. The rumors and misconceptions were from the “Internet Rumor Exposure Station” of the CIDP. In total, 20 health-related rumors and misconceptions were randomly selected using the random number table method. The truths were from “True Knowledge” of the CIDP, an expert-led video platform for debunking rumors. We randomly chose 10 health-related videos. To ensure accuracy, information extraction was completed through discussion by 3 PhD students (YL, YM, and YZ). All samples were randomly inputted into the 2 models in Chinese. Before inputting, the questioning was standardized through group discussion within the research team (YL, YM, and YZ). The questions were conducted in that order. The study process is presented in Figure 1. All the above work has been completed on December 25‐26, 2023. All materials and questions are shown in detail in Multimedia Appendix 1.

### Evaluation

#### Accuracy

Accuracy was assessed by the rate of correctly identifying health-related rumors, misconceptions, and truths. The criterion is whether the model accurately classifies information as a rumor, misconception, or truth in its response. Considering the high similarity between rumors and misconceptions in practice, this study did not overly distinguish between them. As long as the response raised doubts and the study group deemed the doubts reasonable upon discussion, it is considered an accurate judgment.

#### Effectiveness

Effectiveness refers to the degree of correctness in explaining the material. The effectiveness of the explanations was critically appraised. Two researchers (YL and YM) collaboratively searched for relevant evidence, such as the latest relevant guidelines, consensus, standards, or high-quality studies. We compared whether the explanations and recommendations provided by the 2 models align with relevant evidence.

#### Readability

Readability refers to the ease with which a reader can understand a written text. Readability was calculated by the formula [17]: *R* = 17.5255 + 0.0024*X*_1_ + 0.04415*X*_2_ − 18.3344 (1 − *X*_3_), where *R* represents the readability level of the health information. The smaller *R* value indicates that the health essay is easier to read, and the range of *R* value represents reading grade level. For example, 2≤*R*<3 represents the material that was suitable for grades 2-3. *X*_1_ represents the total number of words in the material, excluding punctuation. *X*_2_ represents average sentence length, which is the ratio of total word number to complete sentence number (a complete sentence is a sentence that ends with a period, question mark, or exclamation point). *X*_3_ represents the percentage of medical-related terminologies, calculated as the total number of terminology words divided by the total number of words. The inclusive criteria for medical terminology is the Chinese Medical Subject Headings [18]. The subject headings and subheadings were considered as the medical terminology. Meantime, Chinese Medical Subject Headings is not updated simultaneously, so we further check out the SinoMed and PubMed MeSH Database as the complementary medical terminologies. Additionally, we considered the real-world use of some words commonly understood in daily life, such as “sleep,” “health,” “disease,” “death,” “nutrition,” “medicine,” “vitamin,” and “infection,” and some common diseases including “hypertension,” “diabetes,” and “hyperlipidemia.” Because the readability formula was suitable for persons with primary education [17], the above words were considered as everyday words. All medical terminologies in doubt were resolved through group discussion.

#### Applicability

Applicability refers to the suitability and feasibility of using the material for health science popularization. Applicability was evaluated by the Chinese Suitability Assessment of Materials (CSAM). The interrater reliability of the tool was 0.85, indicating high consistency among raters. Additionally, based on item response theory and generalizability theory, the tool demonstrated good structural validity and discriminant validity [19]. Six dimensions were assessed: content, literacy demand, graphics, layout or typography, learning simulation, and cultural appropriateness. In total, the 22 items were scored on a 3-grade rating from 0 to 2. A total score was from 0 to 44, which showed the better suitability in a higher score, and a percentage (actual to total score) is determined with the results interpreted as follows: 70%-100% as very applicable (grade 1), 40%-69% as applicable (grade 2), and 0%-39% as not applicable (grade 3) [20-22]. In this study, the 2 GLLMs could provide text-only health material without graphics. Finally, 17 items in 5 dimensions were used to evaluate the material except the graphics part, with a total score of 34.

One researcher (YL) randomly sequenced the health essays. Two raters (YZ and JL) engaged in discussions to determine the final evaluation results. They were uncertain which GLLM had generated which health essay.

### Analysis

SPSS (version 26.0; IBM Corp) was adopted to establish the cross-checking database by 2 reviewers (YL and YM) independently. Continuous data were expressed as mean and SD, and categorical data were expressed as frequency or percentage. According to the P-P plot test, the readability and applicability scores showed an approximately normal distribution. A 2-tailed *t* test was adopted to compare the differences in readability scores and applicability scores between the 2 models, and a chi-square test was used to compare the applicability grade. Further, a rank-sum test was selected to compare grade distribution differences among all items of the CSAM between the 2 models. Meanwhile, textual data (health essays) were qualitatively analyzed to further illustrate the differences between items. In addition, we selected the inductive content analysis [23] to summarize the characteristics of the health essays.

According to related studies of the 2 GLLMs, asking questions in Chinese and English or repeating the same question twice may yield different answers [24,25]. We further made postanalysis validation to analyze the stability of the 2 GLLMs. If the first response of GPT-4 is inaccurate, we will rephrase the question in English and ask again. Meanwhile, we repeated the same questions for both models to evaluate reproducibility. According to the related studies [26,27], depending on the size of the dataset, 10%‐25% of data units would be typical. Although there is little consensus regarding the proportion of the data, we selected 25% of cases (5 rumors, n=20 and 3 truths, n=10).

## Results

### Accuracy

For GPT-4, the accuracy rates were 100% (20/20) for identifying rumors or misconceptions and 70% (7/10) for truths. Meanwhile, ERNIE Bot 4.0 achieved 100% accuracy rates for both rumors or misconceptions (20/20) and truths (10/10).

Among the cases not correctly identified, GPT-4 demonstrates hallucinations, revealed as inconsistencies between explanations and judgments [28,29]. For another example, “Drinking milk regularly can still lead to Vitamin D deficiency” is a truth. The outputs of GPT-4 demonstrated that drinking milk alone might not be enough to meet the body’s needs, but it thought this information was a rumor. Similarly, inaccuracies occurred in the identification of other truths.

### Effectiveness

Among the explanations, GPT-4 and ERNIE Bot 4.0 mostly provided widely recognized viewpoints. It is worth noting that the 2 models may provide criteria or indicators of health. For example, GPT-4 recommends an average daily water intake of about 2‐3 L for adults without any additional restrictions. ERNIE Bot 4.0 suggests that in mild climates, persons with light levels of physical activity need to drink 1500-1700 mL of plain water per day. In high temperatures or high levels of physical activity, a moderate increase in water intake is required. In addition, drinking water should be evenly distributed throughout the day to avoid excessive drinking at one time. Furthermore, it also stated that these recommendations come from the Chinese Dietary Guidelines, while the GPT-4 did not provide reference information. We further verified that the citation by ERNIE Bot 4.0 followed the recommendations of the guideline [30]. In response to more specific questions, some suggestions were vague and unclear. For the explanation of “Diabetic patients can eat fruit,” GPT-4 only provided a general principle—controlling blood sugar levels—while ERNIE Bot 4.0 provided more specific requirements—smooth control of postprandial blood glucose within 10 mmol/L—which is in compliance with guidelines [31,32].

### Readability

The readability scores of 30 health essays are presented in Table 1. The average *R* value for the health essays was 2.92 (SD 0.85) in GPT-4 and 3.02 (SD 0.84) in ERNIE Bot 4.0. There was no statistically significant difference between the 2 models (*P*=.65). For the total number of words, ERNIE Bot 4.0 produced longer essays than GPT-4 (*P*=.03). For the average sentence length, ERNIE Bot 4.0’s sentences were shorter than GPT-4’s (*P*<.01). For comparisons of rumors or misconceptions and truths, respectively, please see Tables S1 and S2 in Multimedia Appendix 2.

**Table 1.** The readability scores of health essays generated by 2 generated large language models (n=30).

Number^a^	*X*_1_ ^b^		*X*_2_ ^c^		*X*_3_ ^d^		*R* ^e^	
Bot 1^f^	Bot 2^g^	Bot 1	Bot 2	Bot 1	Bot 2	Bot 1	Bot 2
1	550	531	30.56	31.24	0.06	0.07	2.96	3.19
2	461	497	28.81	26.16	0.09	0.05	3.12	2.39
3	500	445	31.25	26.18	0.05	0.08	2.65	2.90
4	566	574	33.29	26.09	0.07	0.07	3.25	3.03
5	544	501	28.63	26.37	0.03	0.02	2.34	1.89
6	542	619	36.13	32.58	0.02	0.06	2.36	3.12
7	565	552	33.24	30.67	0.10	0.06	3.80	3.03
8	557	457	30.94	20.77	0.07	0.05	3.15	2.13
9	453	690	25.17	32.86	0.09	0.10	3.07	4.13
10	570	696	31.67	32.26	0.13	0.11	4.28	4.24
11	490	439	37.70	32.87	0.06	0.08	3.12	3.24
12	488	423	28.71	24.88	0.05	0.09	2.61	2.87
13	454	588	26.71	29.40	0.06	0.10	2.51	3.68
14	480	502	26.67	27.89	0.17	0.14	4.54	4.26
15	419	535	29.93	28.16	0.07	0.11	2.79	3.64
16	526	554	35.07	26.38	0.15	0.16	4.83	4.56
17	434	490	36.17	27.22	0.03	0.04	2.29	2.24
18	465	530	33.21	24.09	0.03	0.06	2.37	2.67
19	494	460	30.88	27.06	0.03	0.07	2.33	2.80
20	512	504	34.13	29.65	0.16	0.19	4.86	5.20
21	479	478	26.61	28.12	0.03	0.05	2.05	2.54
22	520	677	30.59	30.77	0.00	0.02	1.79	2.45
23	641	534	30.52	23.22	0.01	0.07	2.19	2.80
24	496	415	33.07	24.41	0.01	0.02	1.99	1.58
25	433	804	28.87	27.72	0.07	0.02	2.82	2.73
26	405	632	27.00	31.60	0.10	0.06	3.12	3.23
27	487	752	30.44	30.08	0.01	0.00	1.78	2.32
28	495	384	30.94	20.21	0.03	0.07	2.34	2.25
29	536	569	35.73	28.45	0.02	0.01	2.40	2.01
30	528	688	33.00	29.91	0.11	0.07	3.83	3.44
Average (SD)	503.00 (52.09)	550.66 (104.54)	31.19 (3.21)	27.91 (3.36)	0.06 (0.05)	0.07 (0.04)	2.92 (0.85)	3.02 (0.84)
*t* test (*df*=29)	2.235	2.235	−3.864	−3.864	0.618	0.618	0.460	0.460
*P* value	.03	.03	<.001	<.001	.47	.47	.65	.65

a Numbers 1-20: health science popularization essays based on rumors. Numbers 21-30: health science popularization essays based on truths.

b *X*_1_: total number of words.

c *X*_2_: average sentence length.

d *X*_3_: percentage of medical-related terminologies.

e *R*: readability score.

f Bot 1: GPT-4.

g Bot 2: Enhanced Representation through Knowledge Integration Bot 4.0.

### Applicability

The CSAM scores of 30 health essays are presented in Table 2, and the grade is presented in Table 3. Except for the content and cultural appropriateness, significant differences were observed in the total score and scores in other dimensions between the 2 GLLMs (*P*<.05). Generally, the scores of ERNIE Bot 4.0 were higher than those of GPT-4 except for layout or typography. Among the CSAM grades, significant differences were only found in the learning stimulation dimension (*P*=.003). The item score grade distribution is presented in Table 4 and Multimedia Appendix 3. Significant grade distribution differences were observed in items b3, b5, d3, and e2 (*P*<.05).

**Table 2.** The CSAM^a^ score of health essays generated by 2 GLLMs^b^ (n=30).

Dimension	Score		*t* test (*df=*29)	*P* value
Bot 1^c^, mean (SD)	Bot 2^d^, mean (SD)
Content	7.47 (0.50)	7.70 (0.47)	−1.855	.07
Literacy demand	7.87 (1.04)	8.57 (0.68)	−3.084	.003
Layout or typography	3.53 (0.82)	2.97 (0.93)	2.507	.02
Learning stimulation	3.63 (1.03)	4.33 (0.71)	−3.056	.003
Cultural appropriateness	3.40 (0.56)	3.57 (0.50)	−1.208	.23
Total	25.90 (2.29)	27.13 (1.94)	−2.247	.03

a CSAM: Chinese Suitability Assessment of Materials.

b GLLM: generated large language model.

c Bot 1: GPT-4.

d Bot 2: Enhanced Representation through Knowledge Integration Bot 4.0.

**Table 3.** The CSAM^a^ grade of health essays generated by 2 GLLMs^b^ (n=30)^c^.

Dimension	Bot 1^d^			Bot 2^e^		
	Grade 1	Grade 2	Grade 3	Grade 1	Grade 2	Grade 3
Content^f^	30	0	0	30	0	0
Literacy demand^f^	26	4	0	30	0	0
Layout or typography^g^	0	24	6	0	17	13
Learning stimulation^f^^,^^h^	6	20	4	14	16	0
Cultural appropriateness^f^	29	1	0	30	0	0
Total^f^	26	4	0	29	1	0

a CSAM: Chinese Suitability Assessment of Materials.

b GLLM: generated large language model.

c The percentage (actual to total score) is determined with the results interpreted as follows: 70%-100% as very applicable (grade 1), 40%-69% as applicable (grade 2), and 0%-39% as not applicable (grade 3).

d Bot 1: GPT-4.

e Bot 2: Enhanced Representation through Knowledge Integration Bot 4.0.

f Fisher exact text.

g Chi-square text

h *P*<.05.

**Table 4.** The CSAM^a^ item score of health essays generated by 2 GLLMs^b^ (n=30)^c^.

Item	Bot 1^d^, mean rank	Bot 2^e^, mean rank	*Z* value	*P* value
a1^f^	30.50	30.50	<0.001	>.99
a2^g^	27.00	34.00	−1.818	.07
a3^h^	30.50	30.50	<0.001	>.99
a4^i^	30.50	30.50	<0.001	>.99
b1^j^	31.00	30.00	−1.000	.32
b2^k^	30.50	30.50	<0.001	>.99
b3^l^	27.00	34.00	−2.316	.02
b4^m^	28.00	33.00	−1.376	.17
b5^n^	26.85	34.15	−2.256	.02
d1^o^	30.50	30.50	<0.001	>.99
d2^p^	30.50	30.50	<0.001	>.99
d3^q^	35.43	25.57	−2.470	.01
e1^r^	28.00	33.00	−1.376	.17
e2^s^	25.37	35.63	−2.850	.004
e3^t^	29.27	31.73	−0.682	.496
f1^u^	30.50	30.50	<0.001	>.99
f2^v^	28.28	32.72	−1.126	.26

a CSAM: Chinese Suitability Assessment of Materials.

b GLLM: generated large language model.

c Mann-Whitney *U* test was used to compare between the 2 GLLMs.

d Bot 1: GPT-4.

e Bot 2: Enhanced Representation through Knowledge Integration Bot 4.0.

f a1: purpose is evident.

g a2: content about behaviors.

h a3: scope is limited.

i a4: summary or review included.

j b1: reading grade level

k b2: writing style, active voice

l b3: context is given first

m b4: vocabulary.

n b5: road signs.

o d1: layout factors.

p d2: typography.

q d3: subheadings used.

r e1: interaction used.

s e2: behaviors are modeled and specific.

t e3: motivation.

u f1: match in logic, language, experience.

v f2: cultural image and examples.

### Characteristics of the Health Essays

#### Use of Subheadings

GPT-4 tends to use subheadings. Among the 30 health essays, GPT-4 incorporated the subheading format in a total of 24 papers, whereas ERNIE Bot 4.0 opted for subheadings in 13 papers. Meanwhile, GPT-4 scored higher on item d3 (subheadings used) of the CSAM, indicating the frequent use of subheadings, without statistical significance (Table 4 and Multimedia Appendix 3).

#### Interaction Mode

Two GLLMs prefer to ask and answer themselves and sometimes write lengthy explanatory or illustrative texts that lack subheadings. In health science popularization, it is not only necessary to give readers the correct answers but also to constantly stimulate readers to think [33]. Meanwhile, ERNIE Bot 4.0 scored higher than GPT-4 in item e1 (interaction used) of the CSAM without statistical significance (Table 4 and Multimedia Appendix 3).

#### Linguistic Habits

The advantage of the 2 GLLMs is their preference for the active voice. In Chinese, active voice typically conforms to linguistic conventions. Comparatively speaking, simple sentences are more commonly used in the responses of ERNIE Bot 4.0, while GPT-4 tends to use compound sentences more often. Therefore, ERNIE Bot 4.0 conforms to Chinese language conventions. Furthermore, GPT-4 generates texts with grammatical problems.

#### Content

First, GPT-4 tends to respond more frequently (13 times) to phrases like “the research showed that...” or “Experts suggest or recommend that...” compared to ERNIE Bot 4.0, which used such phrases less often (4 times). Furthermore, neither GPT-4 nor ERNIE Bot 4.0 provided references or citations for these recommendations.

Second, 2 GLLMs can provide specific cutoff points such as water intake. ERNIE Bot 4.0 stated that these recommendations were from the Chinese Dietary Guidelines, but GPT-4 did not provide the original source. However, it is important to note that even ERNIE Bot 4.0 can sometimes fail to cite its sources adequately. For example, a daily intake of 300 g of cooked white rice may serve as a cutoff point, above which the risk of developing type 2 diabetes increases by 13% with every additional intake of 158 g. While this statement appears comprehensive, the lack of an original source leaves us with no way to verify the conclusion.

### Postanalysis Validation

Considering the potential impact of language [13,24], 2 weeks later after the first questioning, we asked GPT-4 in English and received its English response for the truths not correctly identified. However, GPT-4 still responded that “Drinking milk regularly can still lead to Vitamin D deficiency” was a rumor, despite the English explanations were accurate. For other truths, GPT-4 still replied “it is a topic of debate.”

When the same question is submitted twice, it is a possibility that the 2 responses generated may not be similar before and after the second submission [25]. Further, we asked the same questions to the 2 models again from January 23 to 24, 2024. Notably, GPT-4 expressed differential opinions in its 2 responses for the same case (“Eating more spinach for iron supplements”). In the first time, GPT-4 argued that the viewpoint was inaccurate, whereas, in the second time, it considered the view correct. The 2 explanations were consistent, both agreeing that the viewpoint was theoretically feasible but had little effect in practice. Overall, among other cases, there was consistency in the 2 time-point responses with some differences in detail. For instance, about a truth—“You can drink milk on an empty stomach”—in comparison with the first response, GPT-4 added an explanation about food culture.

In the second response, ERNIE Bot 4.0 accurately identified the rumor or misconception, and while the 2 explanations were similar overall, the second one was longer and included more details. For the truth that “You can drink milk on an empty stomach,” the second response provided additional information, covering special situations like lactose intolerance. When it came to the rumor that “Sunlight through glass windows can replenish calcium,” the second response not only explained the cause but also offered dietary advice. Interestingly, in debunking the rumor that “Drinking more water is healthier,” the first response mentioned the recommendation of Chinese Dietary Guidelines, but it disappeared in the second response. This inconsistency might suggest randomness in their responses.

## Discussion

### Principal Findings

When comparing GPT-4 and ERNIE Bot 4.0, both performed well in identifying rumors or misconceptions, but GPT-4 demonstrated hallucinations. Notably, both models provided comprehensive explanations. However, they rarely provided supporting citations for their viewpoints. On the CIDP, without proven scientific evidence, it has been recognized as a rumor and could mislead the public. The unconfirmed information is shared on the web as if it were accurate information, sometimes going viral and causing widespread anxiety or leading to misinformation-based beliefs [34].

For recommendations and viewpoints provided by the 2 GLLMs, we further asked them to offer references or citations. Regrettably, the 2 models indicated that they were unable to provide accurate source material, but they sometimes could provide vague references. Upon further verification, we found that the references cited as evidence were either full of mistakes or completely fabricated. This is consistent with previous studies conducted on earlier versions of ChatGPT [35,36]. Our findings suggest that the same problem still persists on GPT-4 and ERNIE Bot 4.0. Especially, the provided numerical values should be carefully examined. The value may be from nonauthoritative reports [25]. Nevertheless, the 2 models usually provide widely recognized viewpoints, and the recommended healthy lifestyles are also aligned with the guidelines. Overall, despite limitations, they can achieve the effectiveness of science popularization for the general public in the situation that there is no health care professional to judge information.

Readability is a critical factor in comprehension, which refers to how well a passage of text can be read and understood by an individual [37]. For low literacy skills, a fifth-grade level or lower is more suitable [38]. Obviously, the essays generated by the 2 GLLMs met the requirements in reading level (Table 1). In our study, although the readability scores of the 2 GLLMs were not statistically different, ERNIE Bot 4.0 generated longer text than GPT-4 (Table 1). For an individual with lower literacy skills, longer text may lead to loss of interest and even cause reading difficulties. The related studies have proved that GLLMs can provide simplified text, but understanding this text still requires a certain level of education [39-41]. Additionally, reading fluency is an essential aspect of the overall reading experience. The grammatical issues in GPT-4 cannot be overlooked, and GPT-4 tends to generate longer sentences than ERNIE Bot 4.0 (*P*<.001; Table 1). This suggests that texts by ERNIE Bot 4.0 may be easier to read. Nevertheless, text easiness showed no influence on trustworthiness, and the public may still blindly believe this content [42]. Considering the randomness of the responses provided by the 2 models, caution should be made when taking their advice.

ERNIE Bot 4.0 was better than GPT-4 in item b3 (context is given first; *P*=.02) and b5 (road signs; *P*=.02; Table 4 and Multimedia Appendix 3). The context is given before new information. We learn new facts or behaviors more quickly when told the context first. Meanwhile, headers or topic captions tell very briefly what is coming next. These “road signs” make the text look less intimidating and prepare the reader’s thought process to expect the announced topic [38]. GPT-4 performed better than ERNIE Bot 4.0 in item d3 (subheadings used; *P*=.01; Table 4 and Multimedia Appendix 3). Longer texts need to be partitioned into smaller chunks. Subheadings can help readers quickly understand the core content of each chunk [38]. In item e2 (behaviors are modeled and specific), ERNIE Bot 4.0 performed better than GPT-4 (*P*=.004; Table 4 and Multimedia Appendix 3). People often learn more readily when specific, familiar instances are used rather than abstract or general concepts [38]. When presented with the health essay “There is no standard for the ideal time of sleep,” GPT-4 explained more about factors affecting sleep duration. In contrast, ERNIE Bot 4.0 dedicated approximately 50% of its response to helping individuals determine the appropriate length of sleep for themselves.

One of GLLM’s strengths is its ability to sift through massive amounts of information and produce responses in a conversational and easy-to-understand manner. In contrast, search results from current search engines can often be overwhelming for the general public, frequently filled with irrelevant or misleading information [25]. However, as ChatGPT and ERNIE Bot are specifically designed for conversational dialogue, their responses can be more comprehensible than those found in professional guidelines or literature. The study showed that, after reading popularized papers addressed to a lay audience, lay readers agreed more with the knowledge claims presented in those papers and were more confident in their claim judgments than after reading papers addressed to expert readers [43]. In our study, we found that the 2 GLLMs provided easy-to-understand information. However, the information might be superficial or nonspecific with errors. Therefore, if someone agrees with the idea because of the model’s response, the misleading information spreads widely on the web possibly [44].

Another key element for health education is stimulation and motivation. Interaction, in particular, plays a crucial role in health education or popularization. We suggest that a questioning format should be used to keep the reader recalling the educational content. In the health essays provided by the 2 models, a question-and-answer format was used, which involved posing a question and then promptly providing the corresponding answer. Even though it is a passive interaction, it is still more effective in reminding readers to focus on the following answers than long explanatory or illustrative texts. Meanwhile, it is recommended that both models successfully break down complex topics. This not only makes the materials more understandable but also allows the readers to explore the logic of cause and effect. When people believe tasks and behaviors are valuable and practicable, they will be motivated to learn [38,45].

This study has some limitations. First, due to time constraints, we were unable to use all the cases in the rumor library. We chose typical recent cases to test their ability as in previous studies. Second, the readability formula is a general tool. In the future, it is worth exploring to develop the specific tool for Chinese medical materials. Third, the CSAM can help you save time and money and improve program effectiveness by selecting or producing materials that the public is likely to pick up, read, understand, and act on. The CSAM cannot substitute for formative research and testing, through which the public confirms that the information is attractive, useful, and persuasive to them. Fourth, the 2 GLLMs do not provide graphics. In many works of scientific popularization, graphic is an important tool to help readers understand. However, with the emergence of GLLMs’ capability to generate graphics, we need to explore whether this function can assist in science popularization further in depth. Fifth, this study was to compare 2 GLLMs. With more and more GLLMs being developed, future research can further explore the applicability of them in the Chinese context. Finally, this study focuses more on the Chinese context. Future exploration will be to include more linguistics experts to jointly discuss the impact of different languages on large language models.

### Conclusions

ERNIE Bot 4.0 demonstrated similar accuracy to GPT-4 in identifying Chinese rumors. Notably, the 2 GLLMs mostly provided widely recognized viewpoints, despite potential inaccuracies and superficiality. When it comes to the health essays, nurses can learn from the linguistic strengths that facilitate readability and comprehension. However, the content needs to be checked by experts for appropriateness and accuracy. Additionally, ERNIE Bot 4.0 aligns well with Chinese expression habits, making it a suitable choice for those seeking an enhanced Chinese reading experience.

## Supplementary material

10.2196/63188 Multimedia Appendix 1 Health rumor, misconception, and truth.

10.2196/63188 Multimedia Appendix 2 The readability scores of the health essays based on rumor, misconception, and truth.

10.2196/63188 Multimedia Appendix 3 The Chinese Suitability Assessment of Materials item grade distribution.

## Abbreviations

CIDP: China Internet Disinformation Platform
CSAM: Chinese Suitability Assessment of Materials
ERNIE Bot: Enhanced Representation through Knowledge Integration Bot
GLLM: generated large language model
