# Supplementary material for: Comparing the Accuracy of Two Generated Large Language Models in Identifying Health-Related Rumors or Misconceptions and the Applicability in Health Science Popularization: Proof-of-Concept Study
Source: JMIR Form Res. 2024 Dec 2;8:e63188. doi: 10.2196/63188 (PMC11627524; doi:10.2196/63188)
Supplement: Multimedia Appendix 1 [file formative-v8-e63188-s001.docx]

| **No.** | **健康谣言** | **Health rumor** |
| --- | --- | --- |
| 1 | 戴眼镜会加重近视 | Wearing glasses can aggravate myopia |
| **2** | **多吃菠菜能补铁** | **Eating more spinach for iron supplements** |
| 3 | 拔智齿，能瘦脸 | Wisdom tooth extraction can slim down your face |
| **4** | **多服用维生素C能预防流感** | **Taking more vitamin C can prevent the influenza** |
| 5 | 眼药水没过保质期就能继续用 | Eye drops can continue to be used before the expiration date |
| 6 | 空腹运动减肥效果好 | Exercise on an empty stomach for better weight loss |
| 7 | 感染新冠不发烧，意味着免疫力更强 | No fever with COVID-19 means a stronger immune system |
| 8 | 吃白米饭会导致糖尿病 | Rice can lead to diabetes |
| 9 | 心跳越慢，身体越好 | Slower heartbeat means better health |
| 10 | 甜味剂导致肥胖 | Sweeteners lead to obesity |
| **11** | **隔着玻璃晒太阳能补钙** | **Sunlight through the glass windows can replenish calcium** |
| 12 | 茶或咖啡类饮品解酒 | Tea or coffee can be an antidote to alcohol |
| **13** | **喝水量越多越好** | **Drinking more water is healthier** |
| 14 | 喝苏打水能治疗高尿酸 | Soda water can treat hyperuricemia |
| 15 | 喝柠檬水会长结石 | Lemon water can cause kidney stone |
| 16 | 脂肪肝是胖人“专属” | Fatty people get fatty liver disease |
| 17 | 孕妇多吃水果宝宝出生后皮肤好 | When pregnant women eat more fruits, the babies are born with good skin |
| **18** | **白头发会越拔越多** | **the more gray hair appears after pulling it out** |
| 19 | 长期戴眼镜，眼睛会凸变成“金鱼眼” | Eyeballs can become bulging when wearing glasses for a long time |
| 20 | 婴幼儿接种了卡介苗就不会再患结核病 | Children vaccinated with BCG will no longer get tuberculosis |

| **No.** | **健康真相** | **Health truth** |
| --- | --- | --- |
| 1 | 冻疮处用热水浸泡无法有效缓解 | Chilblain is not effectively relieved by soaking it in hot water |
| 2 | 经常喝牛奶仍会维生素D缺乏 | Drinking milk regularly can still lead to Vitamin D deficiency |
| 3 | 不吃早餐更容易变胖 | Skipping breakfast makes you more likely to gain weight |
| **4** | **午睡不易过长** | **Sleeping time is not too long at noon** |
| 5 | 糖尿病患者可以吃水果 | Diabetic patients can eat fruit |
| **6** | **空腹可以喝牛奶** | **You can drink milk on an empty stomach** |
| 7 | 仰头玩手机不能预防颈椎病 | Tilting your head up to play smartphone can not prevent cervical spondylosis |
| 8 | 饭后喝酸奶和乳酸菌饮料不能助消化 | Drinking yogurt and lactobacillus drinks after meals does not help digestion |
| 9 | 理想的睡眠时间并没有硬性标准 | No standard for the ideal time of sleep |
| **10** | **电子烟会上瘾** | **E-cigarettes are addictive** |

| **No.** | **标准化提问** | **Standardized Questions** |
| --- | --- | --- |
| 1 | 请判断以下内容是否为谣言或误解： | Please judge if the following statement is a rumor or misconception |
| 2 | 请进一步解释你的判断 | Please provide further explanations of your judgment |
| 3 | 基于以上判断请形成健康科普短文 | Based on the above explanations, please generate a short health science popularization essay |

Note: Bold font – The samples in the Post-analysis validation
