# Supplementary material for: Comparing the Accuracy of Two Generated Large Language Models in Identifying Health-Related Rumors or Misconceptions and the Applicability in Health Science Popularization: Proof-of-Concept Study
Source: JMIR Form Res. 2024 Dec 2;8:e63188. doi: 10.2196/63188 (PMC11627524; doi:10.2196/63188)
Supplement: Multimedia Appendix 2 [file formative-v8-e63188-s002.docx]

**Table S1.** The readability scores of health essays based on rumors (n=20).

|  | **X1** | | **X2** | | **X3** | | **R** | |
| --- | --- | --- | --- | --- | --- | --- | --- | --- |
|  | **Bot 1** | **Bot 2** | **Bot 1** | **Bot 2** | **Bot 1** | **Bot 2** | **Bot 1** | **Bot 2** |
| **Average ^a^** | 503.50±47.11 | 529.35±75.96 | 31.44±3.44 | 28.14±3.27 | 0.07±0.05 | 0.08±0.04 | 3.16±0.85 | 3.26±0.87 |
| **t value** | 1.293 | | -3.117 | | 0.726 | | 0.363 | |
| **P value** | 0.204 | | 0.003 | | 0.472 | | 0.719 | |

Note: X1: total number of words; X2: average sentence length; X3: percentage of medical related terminologies; R: readability score; Bot 1: GPT-4; Bot 2: ERNIE bot 4.0;

a: Average value is presented as mean and standard deviation (M±SD).

**Table S2.** The readability scores of health essays based on truths (n=10).

|  | **X1** | | **X2** | | **X3** | | **R** | |
| --- | --- | --- | --- | --- | --- | --- | --- | --- |
|  | **Bot 1** | **Bot 2** | **Bot 1** | **Bot 2** | **Bot 1** | **Bot 2** | **Bot 1** | **Bot 2** |
| **Average ^a^** | 502.00±63.67 | 593.30±141.44 | 30.68±2.79 | 27.45±3.69 | 0.04±0.039 | 0.04±0.028 | 2.43±0.65 | 2.53±0.55 |
| **t value** | 1.861 | | -2.206 | | 0.101 | | 0.388 | |
| **P value** | 0.079 | | 0.041 | | 0.921 | | 0.703 | |

Note: X1: total number of words; X2: average sentence length; X3: percentage of medical related terminologies; R: readability score; Bot 1: GPT-4; Bot 2: ERNIE bot 4.0;

a: Average is presented as mean and standard deviation (M±SD).
