# Supplementary figures and images for: Comparing the Accuracy of Two Generated Large Language Models in Identifying Health-Related Rumors or Misconceptions and the Applicability in Health Science Popularization: Proof-of-Concept Study
Source: JMIR Form Res. 2024 Dec 2;8:e63188. doi: 10.2196/63188 (PMC11627524; doi:10.2196/63188)

**
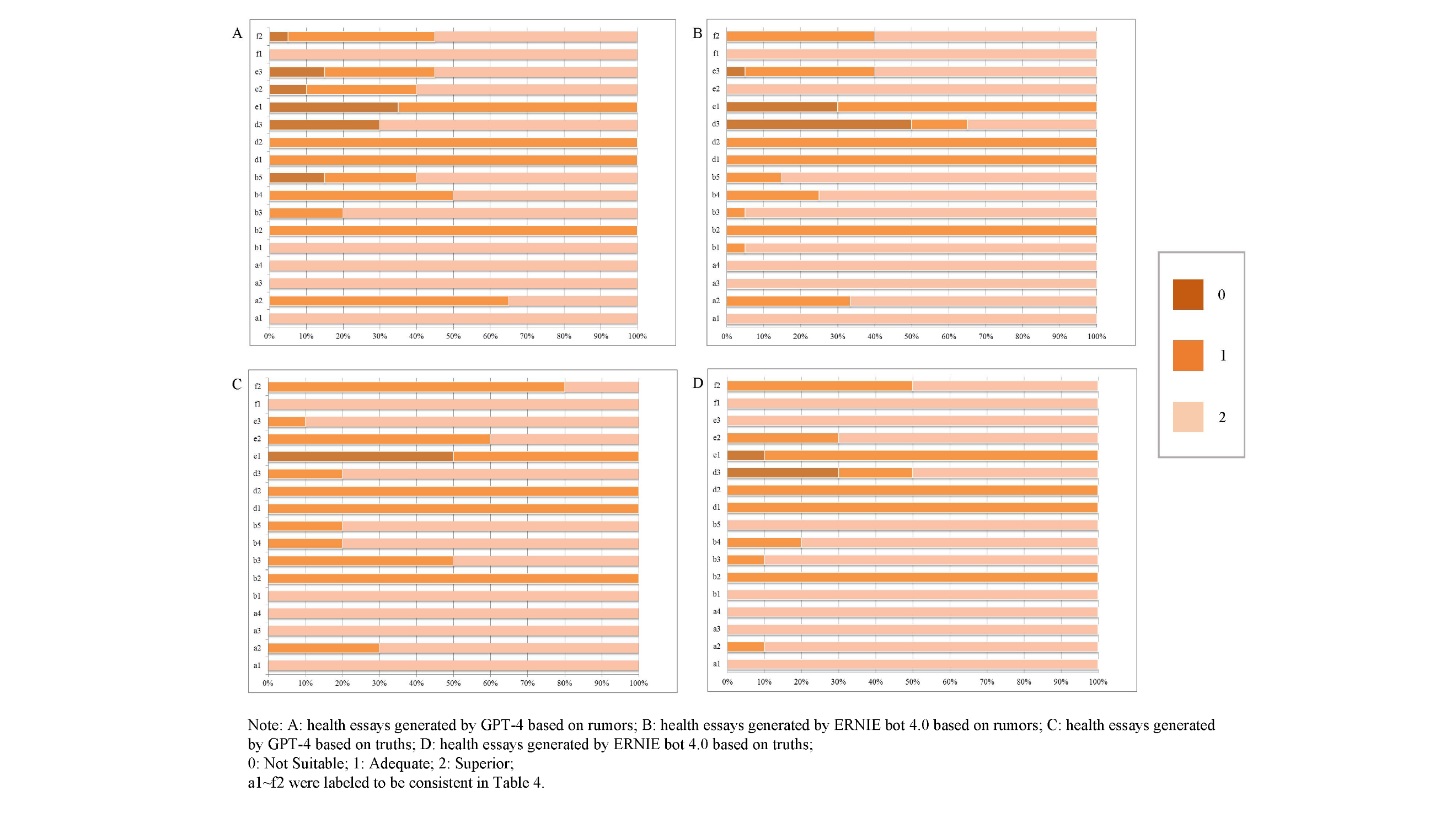
**

Supplement: Multimedia Appendix 3 [file formative-v8-e63188-s003.docx]
